# Large field-induced-strain at high temperature in ternary ferroelectric crystals

**DOI:** 10.1038/srep35120

**Published:** 2016-10-13

**Authors:** Yaojin Wang, Lijun Chen, Guoliang Yuan, Haosu Luo, Jiefang Li, D. Viehland

**Affiliations:** 1 School of Materials Science and Engineering, Nanjing University of Science and Technology, Nanjing 210094, Jiangsu, China; 2 Shanghai Institute of Ceramics, Chinese Academy of Sciences, 215 Chengbei Road, Jiading, Shanghai 201800, China; 3 Materials Science and Engineering, Virginia Tech, Blacksburg, Virginia 24061, USA

## Abstract

The new generation of ternary Pb(In_1/2_Nb_1/2_)O_3_-Pb(Mg_1/3_Nb_2/3_)O_3_-PbTiO_3_ ferroelectric single crystals have potential applications in high power devices due to their surperior operational stability relative to the binary system. In this work, a reversible, large electric field induced strain of over 0.9% at room temperature, and in particular over 0.6% above 380 K was obtained. The polarization rotation path and the phase transition sequence of different compositions in these ternary systems have been determined with increasing electric field applied along [001] direction based on x-ray diffraction data. Thereafter, composition dependence of field-temperature phase diagrams were constructed, which provide compositional and thermal prospectus for the electromechanical properties. It was found the structural origin of the large stain, especially at higher temperature is the lattice parameters modulated by dual independent variables in composition of these ternary solid solution crystals.

Over the past century, material scientists have made great efforts to seek and create new materials with significant mechanical response functionalities for applications in sensing and actuation, ranging from inorganic to organic^1^, metal to nonmetal^2^, and bulk to nano materials^3^,^4^. Of the many types of actuator materials (including magnetostrictive^2^,^5^, photostrictive^6^,^7^, and shape memory alloys^2^,^4^), piezoelectric materials^3^,^8^,^9^,^10^,^11^,^12^,^13^,^14^ are widely used due to their superior high force generation, high frequency capabilities, displacement accuracy, and/or device miniaturization^8^. In particular, relaxor ferroelectric binary single crystals, regarded as generation I^15^,^16^,^17^, i.e., (1 − x)Pb(Mg_1/3_Nb_2/3_)O_3_-xPbTiO_3_ (PMN-PT) and (1 − x)Pb(Zn_1/3_Nb_2/3_)O_3_-xPbTiO_3_] (PZN-PT), with compositions near the morphotropic phase boundary (MPB), exhibit ultrahigh electromechanical coefficients that are up to an order of magnitude larger than that of conventional Pb(Zr_x_Ti_1−x_)O_3_ (PZT) ceramics^8^,^18^,^19^. This important breakthrough^20^ triggered a race for theoretical understanding of the origin of such high responses in perovskite oxides^21^,^22^,^23^,^24^,^25^, and for the experimental identification of the MPB that is a nearly vertical boundary between rhombohedral (*R*) and tetragonal (*T*) phases^26^,^27^,^28^.

In spite of the ultrahigh electromechanical properties of these bianary crystals, several obstacles have restricted them from practical applications^15^,^16^. These includes (i) deterioration in performance with increasing temperature, which limits their operational temperature ranges; and (ii) the coercive field is on the order of 2 kV/cm, which restricts their usage to low power applications. In order to circumvent these difficulties, numerous investigations have been performed, including doping (Mn) binary crystals, crystallization or texture orientation instead of conventional PZT ceramics, and adding solid solution components to achieve ternary systems^15^,^16^.

The challenges associated with property superiority, operational stability, cost and crystal size, have been addressed, in particular after the development of ternary Pb(In_1/2_Nb_1/2_)O_3_-Pb(Mg_1/3_Nb_2/3_)O_3_-PbTiO_3_ (PIN-PMN-PT) single crystals^15^,^16^,^29^,^30^. This generation II system combines the advantages of two respective MPBs, determined by the two individual binary PMN-PT and PIN-PT solid solutions (see Fig. 1(b)). In turn, the MPB zone, rather than a line in the binary systems, offers a higher degree of structural instability and thus provides the possibility to enhance the electromechanical properties^16^. Furthermore, due to complexity in its compositional components, the electromechanical property and Curie temperature (*T*_C_) can be simultaneously optimized and enhanced by varying the concentration of the components near the MPB region, rather than by property tradeoffs as in a binary system (i.e., increase of *T*_C_ at cost of decrease in piezoelectric properties).

However, to date, due to the multiplicity of induced phase transitions with *E* and temperature^31^, the sequence of temperature dependent field-induced strains is not clear for ternary PIN-PMN-PT crystals. This presents a significant challenge to end-users on how to select crystals with different compositions for specific application conditions, especially with regards to operational temperature ranges. In this paper, the electromechanical and piezoelectric properties of PIN-PMN-PT with compositions near the MPB have been systematically characterizated. Large field-induced strains >0.6% in the high temperature range from 360 K to 380 K have been obtained. Furthermore, the field-temperature phase diagrams were established by a comprehensive x-ray diffraction study. The results provide a compositional and thermal prospectus for the electromechanical properties, which can assist end-users to choose compositions for actuation applications at various operational temperature ranges.

## Results and Discussion

### Dielectric and piezoelectric properties

**Figure 1:**
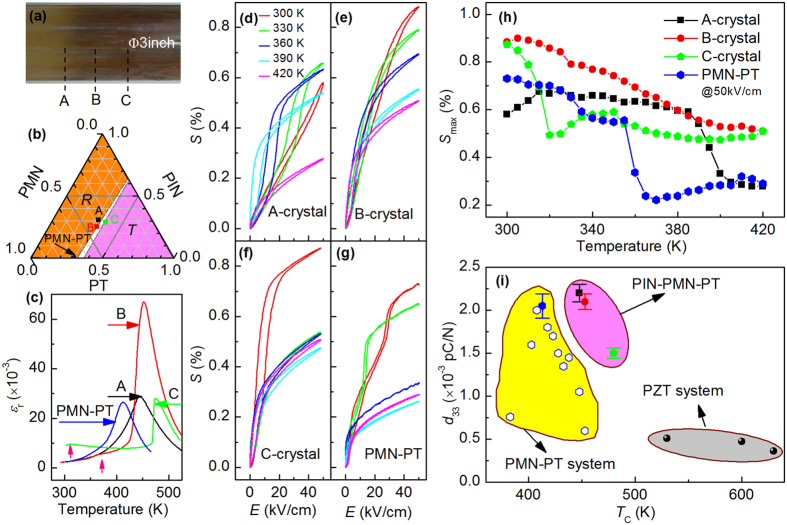
Comparison of piezoelectric and dielectric properties of ternary PIN-PMN-PT and binary PMN-PT systems. (**a**) Photograph for an as-grown large-size PIN-PMN-PT ternary crystal (larger and clearer ones are presented in Fig. S1). (**b**) Phase diagram for these generation II crystals, where the rhombohedral and tetragonal phases are separated by a MPB zone. (**c**) Temperature dependent dielectric constant at 1 kHz in the zero-field-heating condition and unipolar *E*-induced strains for crystals (**d**) A, (**e**) B, (**f**) C and (**g**) PMN-30PT. (**h**) Temperature dependence of the maximum unipolar strains under *E* = 50 kV/cm for the crystals A, B, C, and PMN-30PT. (**h**) Piezoelectric coefficient *d*_33_ of various piezoelectric material systems, including the binary crystal PMN-PT, new generation (A-, B- and C-) ternary crystals, and conventional PZT ceramics (refs 40,41) as a function of *T*_c_. For the PMN-PT system, the *d*_33_ of PMN-30PT was measured in this work, whereas that for other compositions was cited from refs 29,45.

Three typical regions of the as-grown crystal with a nominal composition of 33PIN-35PMN-32PT were selected for investigation (designated as A, B and C), as shown in Fig 1(a). The compositions of the *A*-, *B*- and *C*-crystal were determined to be 31PIN-37.2PMN-31.8PT, 25.4PIN-41.2PMN-33.4PT and 28.8PIN-33.3PMN-37.9PT by quantitative X-ray fluorescence analysis (PHILIPS PW2404 X-ray fluorescence spectrometer) based the standard of In_2_O_3_, MgO and TiO_2_^32^,^33^. All the three crystals are located near MPB, but *A*- and *B*- crystals in the rhombohedral region, while *C*-crystal in the MPB, close to tetragonal region, as schematically illustrated in Fig. 1(b). The ground state of the crystals will be discussed based on the diffraction data. Figure 1(c) shows the dielectric constant (*ε*_r_) as a function of temperature for [001] oriented crystals. The results demonstrate that the binary PMN-30PT has a *T*_C_ near 413 K; whereas the ternary *A*-, *B*- and *C*-crystals have higher *T*_C_ values of 445, 452 and 475 K, respectively. The improvement of the Curie temperature provides potential superiority of ternary crystals to be operated in a wider temperature range. Except for the ferroelectric-paraelectric phase transition peaks, no other additional peaks were apparent for either PMN-30PT or the ternary *A*-crystal; however, additional peaks were distinguishable at lower temperatures for the ternary *B*- and *C*-crystals near 367 and 315 K, respectively. These additional phase transitions will be discussed in more detail below.

The *E*-induced strain (*S*) for (001) oriented crystals is shown in Fig. 1(d–g). These data were taken at various temperatures under a maximum ac electric field (*E*_m_) of 50 kV/cm. For the ternary *A*-crystal, it can be seen that the *S*-*E* curve is nearly linear and anhysteretic at 300 K. On heating to 420 K, the value of *S*_m_ under *E*_m_ = 50 kV/cm increased, reaching a maximum value and then subsequently decreasing. Interestingly, the degree of hysteresis also increased and subsequently decreased. For the ternary *B*-, *C*- and binary PMN-30PT crystals, the *S*-*E* curves approach a saturation near *E*_m_ = 50 kV/cm at all the temperatures studied. It is worth noting that similar results in the ternary ferroelectric crystals with compositions near the MPB were also observed by Zhang *et al*.^34^. Interestingly, diffuse inflections, rather than discontinuous jumps (i.e., first-order-like characteristic^35^) in *S* were found for all the ternary crystals studied, especially at room temperature, which is in contrast to the [001]-oriented binary PMN-30PT crystal that has two abrupt jumps due to the previously reported phase transformational sequence of *M*_A_ → *M*_C_ → T^26^,^36^,^37^,^38^. The atypical *S*-*E* curves for ternary systems might be due to a bifurcated polarization rotation path or diffuse phase transitions with increasing *E*^39^.

The temperature dependence of *S*_m_ at *E*_m_ = 50 kV/cm is summarized in Fig. 1(h). Among the ternary and binary crystals studied, the *B*-crystal was found to exhibit the highest value of *S*_m_ over the temperature range from 300 to 420 K amongst the ternary and binary crystals studied. The value of *S*_m_ for the *B*-crystal near linearly decreased with increasing temperature. Strains as high as *S*_m_ = 0.9% were obtained under *E*_m_ = 50 kV/cm at room temperature for the *B*-crystal, which was 1.5× higher than that of PMN-30PT. Note that the enhancement was even larger for the *B*-crystal if higher fields were applied, as its *S*-*E* curve did not completely saturate (see Fig. 1e). In contrast, the other crystals exhibited discontinuous decreases in *S*_m_ with increasing temperature: A-crystal near 315 and 385 K, *C*-crystal near 320 and 350 K, and the binary PMN-30PT near 320 and 340 K (see Fig. 1h). These abrupt changes are indicative of phase transformations, as will be discussed based on x-ray diffraction below.

Comparing the results in Fig. 1(h), it was found the significance was not only the enhancement of *S*_m_ in the ternary crystals at room temperature, but also the large value of *S*_m_ can be achieved at high temperature range, especially for *B*-crystal, relative to the binary PMN-30PT ones. Obviously, all the ternary crystals exhibit a large value of *S*_m_ > 0.5% over the moderate high temperature range, while PMN-30PT have a much lower value <0.3% above 365 K. In particular, the value of *S*_m_ of *B*-crystal is ×2.8 larger than that of PMN-30PT around 365 K, and ×1.8 larger at 420 K. These results reveal that the new generation ternary crystals, especially the *B*-crystal, have ambiguous preponderance in actuator applications over high temperature range.

With regards to practical applications, the piezoelectric coefficient *d*_33_ and the value of *T*_C_ are two key figures of merit, which significantly depend on crystal composition. Figure 1(i) shows the values of *d*_33_ for various piezoelectric material systems as a function of their *T*_C_. It can be seen that the value of *d*_33_ for PMN-PT crystals with a composition near MPB can reach above 2000 pC/N, while their value of *T*_C_ is near 410 K. Development of higher *T*_C_ values for binary crystals (i.e, doping Mn), unfortunately, cause a significant decrease in the piezoelectric constant, which is another technical obstacle for their practical applications in high-power actuators. The *T*_C_ of conventional piezoceramics, such as PZT, is much higher than that of the binary crystals, but unfortunately, the piezoelectric constant was found to be on the order of 200–700 pC/N^40^,^41^. However, for the generation II ternary crystals, the *d*_33_ value is comparable to (and even higher than) that of the binary crystals, but their *T*_C_ values are several tens degree higher than that for the binary crystals. For the *A*- and *B*-crystal, the value of *d*_33_ was greater than 2000 pC/N, and their *T*_C_ was between 450 and 455 K. The *C*-crystal has a significantly higher *T*_C_ of about 480 K, with a *d*_33_ value of about 1500 pC/N. These results demonstrate the superior characteristics of new generation II crystals in high-temperature range applications.

### Electric field-temperature phase diagram

**Figure 2:**
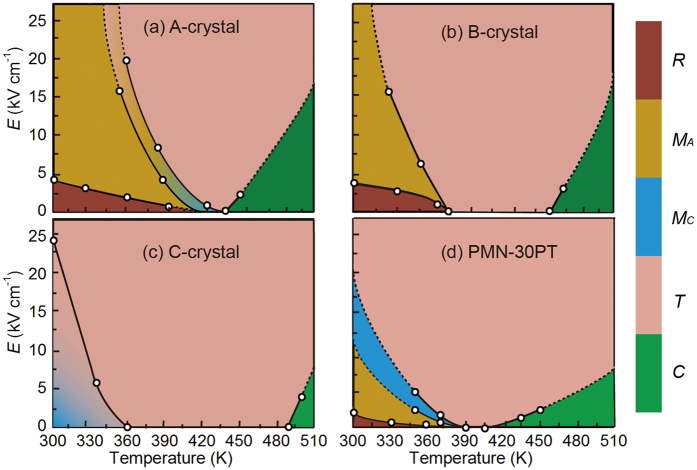
Electric field-temperature phase diagram for the ternary A-, B- and C-crystals, and the binary PMN-30PT crystal. The data of the binary PMN-30PT crystal was adapted from ref. 38. The open circles represent the phase transformational temperatures and fields that were determined from high-resolution x-ray diffraction measurements. These data points were obtained on *in situ* increasing electric-fields and temperatures. Both the solid and dash lines are to guide the eyes. The regions in different colors indicate the different phase fields, and regions of phase coexistence are designated by color mixture.

In order to better understand the structural origin of the temperature dependence of *S*_m_, x-ray diffraction was performed on *A*-, *B*- and *C*-crystals for various thermal and electrical histories. Based on these diffraction data, electric field-temperature diagrams for the ternary crystals, together with that of the binary PMN-30PT one, have been established, as shown in Fig. 2. This summary is presented first for the convenience of the readers: the raw data, including line and reciprocal-space mesh scans (RMS) will be given below and in the supplementary document. Circles designate the phase transformational temperatures and fields, obtained by increasing field beginning from the annealed state at each typical temperature.

### X-ray diffraction data and phase transition sequence

**Figure 3:**
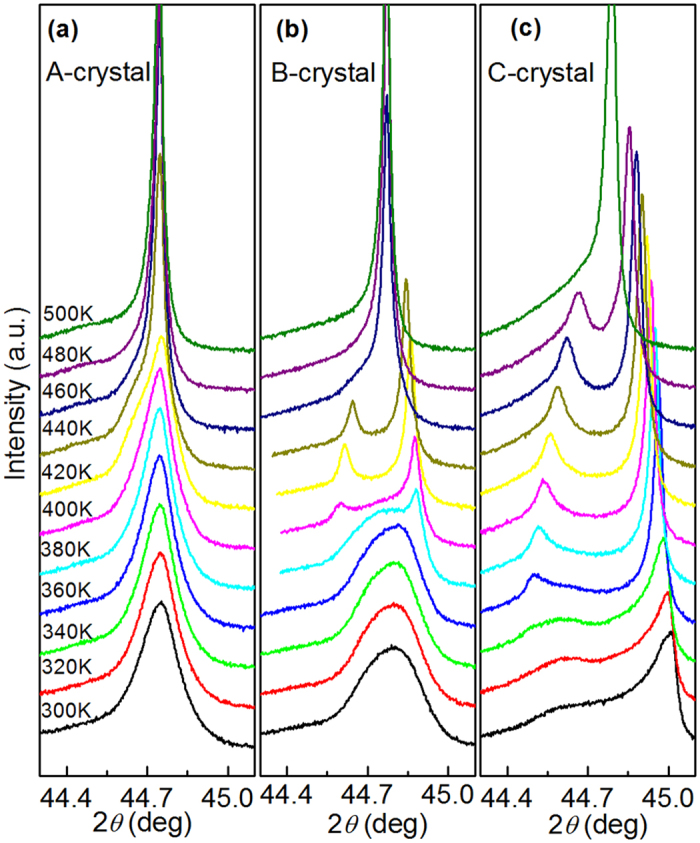
Evolution of diffraction peaks along the pseudocubic (002) zone with temperatures in the zero-field-heating condition for A-, B- and C-crystals. Measurements were from the annealing state.

Figure 3 shows the evolution of line scans around the (002)_C_ zone of ternary *A*-, *B*-, and *C*-crystals with temperature on zero-field-heating condition. Scans were obtained at 20 K intervals beginning from the annealed state. Only a single diffraction peak was observed for the *A*-crystal over the entire measurement temperature range (Fig. 3a). The exception was the presence of weak humps on the low 2θ side at temperatures 420 and 440 K, indicating a weak tetragonal (002)_T_ phase may coexist with the *R* and *C* phases, analogous to that previously reported in binary crystals at low PT contents^27^. For the *B*-crystal, a broad single peak was found below 360 K; but on heating to 380 K, an additional peak appeared on the high 2*θ* side. On further heating above 400 K, the initial broad peak gradually vanished and a doublet peak became evident. This line profile evolution reveals a *R* → *T* transition near 380 K, which agrees well with the discontinuity observed in the *ε*_r_-temperature curve (see Fig. 1c). Note that the initial broad peak is asymmetric, where a weak tail existed at the lower 2*θ* side. This peak tail may be due to phase coexistence, as the *B*-crystal was quite close to the MPB. After heating to 460 K, a subsequent *T* → *C* transition was found. This is evidenced by the appearance of a single cubic (002) peak, and the simultaneous disappearance of the tetragonal (200)-(002) doublet splitting. For the *C*-crystal, a tetragonal (200)_T_ peak of significant intensity was observed below 480 K, and a *T* → *C* phase transition was evidenced at 500 K as the tetragonal (200)-(002) doublet merged into a sharp cubic (002)_C_ singlet. At temperatures below 340 K, in addition to the tetragonal (200)_T_ peak, a broad tail that extended to lower values of 2*θ* was found. This is consistent with its composition being near to the MPB region but on the tetragonal phase. Phase coexistence near and below 340 K is a probable explanation for these line profile charactertics below 360 K, and to its *ε*_r_-temperature curve (see Fig. 1c), which is similar to that previously reported for PMN-34PT crystals^42^.

**Figure 4:**
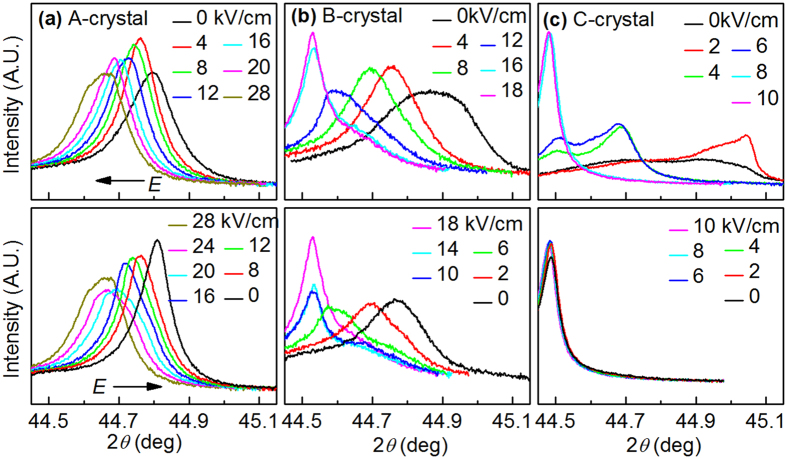
Line scans along the pseudocubic (002) zone for A-, B- and C-crystals at 330 K. In each figure, the top and bottom panel presents the evolution of the diffraction peaks with *in situ* increasing and decreasing *E*//[001], respectively.

In order to provide a link between the macroscopic strain characteristic and the evolution of the structure, comprehensive x-ray diffraction experiments were performed on the ternary crystals with *E* applied *in situ* along the [001] direction. Figure 4 shows the evolution of the diffraction peaks around (002)_C_ zone with increasing and decreasing *E* using field step of 2 kV/cm (typical results shown here). These data were taken at a temperature of 330 K. Additional results at other temperatures are given in Figs S2–4. It is obvious that the peaks shift towards lower values of 2*θ* with increasing *E* (top panel), while shifting back towards higher value of 2*θ* with decreasing *E* (bottom panel).

For the A-crystal, a singlet diffraction peak was found that gradually moved with increasing *E* up to 20 kV/cm, which thereafter split into three peaks at *E* = 28 kV/cm. Analogous to prior reports of binary crystals under *E*//[001]^36^,^37^,^38^,^43^, it can be conjectured that *M*_A_, *M*_C_ and *T* phases coexist; correspondingly, these three peaks can be indexed as (002)_T_, (002)_*M*C_ and (002)_*M*A_ from low to high 2*θ*. A singlet remained and gradually shifted to lower 2*θ* values with increasing field between 0 ≤ *E* ≤ 12 kV/cm for the *B*-crystal. For *E* = 12 kV/cm the singlet was splitted into two peaks of comparable intensities, indicating a region of two-phase coexistence. With further increase of *E*, a single sharp peak became apparent, which was nearly insensitive to further increase of *E* (see Fig. 4b). These features unambiguously show that a *T* phase appears.

The annealed *C*-crystal had a complex diffraction profile whose diffraction peaks were difficult to resolve. This may be due to being near the MPB region but on the *T* phase. Upon application of *E*//[001], the diffraction peaks became considerably more distingushable, as can be seen in Fig. 4(c). In this case, a strong asymmetrical peak was found that had a tail at lower 2*θ* values under *E* = 2 kV/cm. These results demonstrate that the c-axis of the monoclinic phase is rotated and fixed along the *E* field direction, and that a low volume fraction of tetragonal *c*-domain may coexist with it that are also aligned parallel to *E*. With further increasing *E*, the monoclinic phase gradually transformed into the *T* one, as manifested by the relative intensity of doublet (i.e., (002)_T_-(002)_M_) under *E* = 4 and 6 kV/cm. For *E* > 8 kV/cm, a single c-domain *T* phase was obtained. It is interesting to note that this tetragonal single c-domain state was metastable after removal of *E*, as no diffraction intensity corresponding to the monoclinic phase was apparent near 44.7°, in contrast to that observed at 300 K (see Fig. S4a). At higher temperatures (i.e., 360 K), the metastable single c-domain state was partially restored to a multi-domain state upon removal of *E*, at least a small fraction of a-domains was found near 44.9° due to depoling (see Fig. S4b). This diffraction feature reveals a structural origin for the noticable decrease in *S*_m_ near 320 K (Fig. 1g), and a slight increase with increasing *E* thereafter. Changes in *S*_m_ with temperature above 350 K was also observed, which can be explained by a tradeoff between depoling effects and a temperature dependent c/a ratio (see Fig. 3c): i.e., with increasing temperature, the fraction of a-domains increased, but the strain contribution from the unequal crystallographic axis switching decreased.

**Figure 5:**
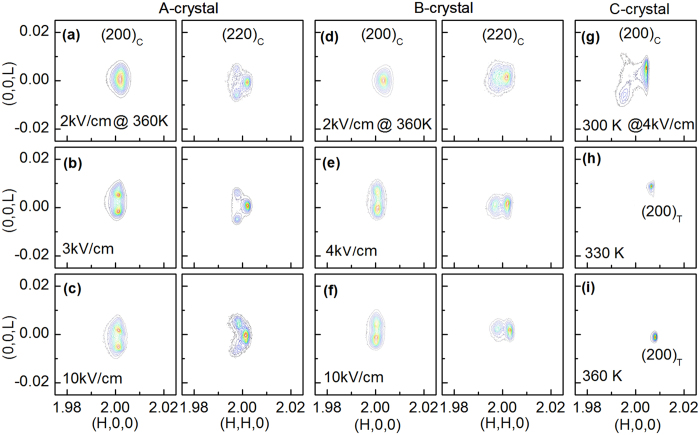
(**a–f**) Evolution of reciprocal-space mesh scans along the pseudocubic (200) and (220) zones for A- and B-crystals at 330 K with increasing *E*. (**g–i**) Evolution of reciprocal-space mesh scans along the pseudocubic (200) for C-crystals under *E* = 4 kV/cm at typical temperatures.

To clarify and determine the phase transformation sequence in Fig. 2, reciprocal-space mesh scans were performed about the (200)_C_ and (220)_C_ zones at various temperatures with *E*//[001]_C_ for A-, B- and C-crystals, as shown in Fig. 5 and S5–S9. For the A-crystal, the *R* phase was evidenced by a broad peak around the (002)_C_ (see Fig. S5a), and a doublet about (220)_C_ (see Fig. S6a) under small *E*. Above 4 kV/cm, the pseudocubic (220) reflection exhibited the characteristic triple splitting of the *M*_A_ phase, which consists of a (040)_MA_ *b*-domain and two twined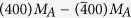 *a*-domains. The (200)_C_ zone exhibited the signature doublet splitting consisting of two twined 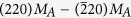 domains. At 330 K, a similar domain configuration evolution and phase transformational sequence was obtained, but the *R* → *M*_A_ transition occurred at a lower field of *E* = 3 kV/cm, as shown in Fig. 5b. Note that at *E* = 2 kV/cm, only a broad single peak with a small splitting along the transverse direction was found around (200)_C_, while a weak triplet was observed around (220)_C_. With increasing field to *E* = 10 kV/cm, a complex domain configuration was found: which consisted of a strong peak around (220)_C_ accompanied by weak diffraction tails (see Fig. 5c). Moreover, line scans around (002)_C_ revealed a single peak with a weak tail on the low 2*θ* side. These abnormal domain configurations can be attributed to coexistence of *M*_A_ and *M*_C_ phases by referring to the polarization rotation pathways for binary crystals^26^,^37^,^38^. Complex domain configurations were also observed under a moderate field of *E* = 2 kV/cm at 390 K. These results indicate the coexistence of *M*_A_ and *M*_C_ phases, in particular in consideration of the observation of a doublet in the (002)_C_ line scans. At 420 K, a single domain configuration along both (200)_C_ and (220)_C_ became sharp under *E* > 0.5 kV/cm, and the lattice parameters extracted from line scans revealed that the structure is tetragonal.

For the *B*-crystal, the *R* phase was stable under moderate field level, as evidenced by a broad diffraction peak around (002)_C_ and a doublet-like feature about (220)_C_ (see Fig. 5d, S7a and S8a). On application of higher *E*, the (200)_C_ diffraction split into two peaks (i.e., *E* = 4 kV/cm at 300 and 330 K, and *E* = 2 kV/cm at 360 K, as shown in Fig. 5d, S7a,b). These domain configurations reveal a *R* → *M*_A_ transition with increasing *E*. It is important to note that atypical domain configurations around (220)_*C*_ were found in the *M*_A_ phase (see Fig. 5e): a *b*-domain and only a single a-domain was observed, rather than a *b*-domain containing two *a*-domains as in previous studies^26^,^37^,^38^. Absence of diffraction peaks can be common in ferroelectric crystals due to defects, or internal stress, in the crystals that break domain equivalency^10^. With increasing *E* to 10 kV/cm, a phase transition of *M*_A_ → *T* was found near 360 K, which was manifested as a sharp single domain with a slight tail around (220)_C_ (see Fig. S7b), where the twined doublet 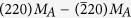
was merged into a sharp single (200)_T_ that had undistinguishable tails. At 390 K, the reciprocal-space mesh scans provide conclusive evidence of the coexistence of *R* and *T* phases at *E* = 0 kV/cm: strong (200)_T_ and (002)_R_ can be seen in Fig. S7c. Upon application of *E* > 2 kV/cm, the two phase coexistence disappeared, and the system transformed into a single domain as evidenced by singlets about both (200)_C_ and (220)_C_.

For the *C*-crystal, the evolution of the line scans and phase transition sequence with increasing *E* were easier to distinguish relative to the *A*-and *B*- crystals (see Fig. 4c). Therefore, the reciprocal-space mesh scans were performed under *E* = 4 kV/cm at various temperatures, as shown in Fig. 5(g–h) and S9. At 300 K, the (200)_C_ and (220)_C_ Bragg peaks split along the longitudinal direction, demonstrating a coexistence of monoclinic and tetragonal phases (see Fig. 4c). As can be seen in Fig. 5(g) and S9(a), a single strong peak coexisted with a weak triplet, i.e., (200)_T_, (020)_*M*C_ *b*-domain and two twined 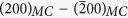
*a*-domains. This reveals a predominant tetragonal phase with a small fraction of the *M*_C_ phase. The domain configuration along (220)_C_ was more complex, however, a single strong peak was obvious, corresponding to the (220)_T_ domain, where the lattice parameter is consistent with that for (200)_T_. At higher temperatures (i.e., 330 and 360 K), a pure *T* phase field was obtained on application of *E* = 4 kV/cm.

Finally, the structural origin of large strain for ternary crystals at high temperature range can be understood by the electric field-induced phase transitions (see Fig. 2). Based on the symmetry-allowed polarization rotation theory, a final state of single tetragonal *c*-domain can be approached under application of large *E*-filed parallel to the polar vector of tetragonal phase^21^. For crystals with compositions near the MPB, the electric field induced strain was mainly attribtued to lattice change due to the phase transition from *M*_A_ → *M*_C_ and *M*_C_ → *T*^26^,^31^,^36^,^44^. As discussed in Fig. 1h, the value of *S*_m_ for PMN-30PT crystals obviously decreased at 330 and 365 K, and was ~0.3% over the temperature above 365 K. Based on the phase diagram, it can be conjectured that the first dropping of *S*_m_ at 330 K was resulted from the temperature induced phase transition of *M*_A_ → *M*_C_, resulting in the vanishing of strain contribution via *E*-filed induced unit cell distortion of *M*_A_ to *M*_C_, while the second one at 365 K was due to the temperature induced phase transition of *M*_C_ → *T*, leading to the disappearance of strain contribution from the polarization rotation from monoclinic plane to [001]. In turn, the strain of PMN-30PT crystals at high temperature range (i.e., above 365 K) was mainly from the polarization extension of tetragonal *c*-domain. Nevertheless, the *B*-crystal was still in the *R*-phase field at temperature around 365 K, indicating the main strain contribution is polarization rotation, i.e, *M*_A_ → *T* phase transition. At temperature above 390 K, even thought the *B*-crystal in *T*-phase field, the diffraction data shows that it was in multi-domain state, rather than a single tetragonal c-domain in PMN-30PT^38^: a sharp tetragonal c domain was remained, which was coexisted with a weak tetragonal a domain at 390 K (see Fig. S3c). These multi-domain states are the structural origin of *B*-crystal exhibiting a much larger strain than PMN-30PT at higher temperature region. In other words, again, under *E*//[001], the strain in *B*-crystal was contributed from the polarization rotation and domain switching, rather than a polarization extension in PMN-30PT at this high temperature region.

## Conclusions

In conclusion, a reversible, large *E*-field induced strain of over 0.9% at room temperature, and in particular over 0.6% above 380 K, was observed in a new generation of ternary piezoelectric single crystals. The polarization rotation path and the phase transition sequence of different compositions in these ternary systems have been determined with increasing *E* applied along [001] direction based on x-ray diffraction data. Electric field-temperature phase diagrams were then constructed. For crystals in the *R* phase field, a composition-induced bifurcation of the polarization rotation path was observed, and in particular, in contrast to binary PMN-30PT, no *M*_C_ phase (or at least no single phase) was found during the *E*-field induced phase transition sequence. The large strain in ternary crystals, especially at higher temperature, is attributed to the lattice parameters modulated by dual independent variables in composition of these ternary solid solution crystals.

## Methods

### Specimen preparation

High-quality and large size ternary crystals with nominal composition of 33PIN-35PMN-32PT were grown by a modified Bridgman method^29^ using high purity (better than 99.99%) PbO, In_2_O_3_, MgO, Nb_2_O_5_ and TiO_2_ as starting materials. Due to composition segregation, the real composition of the crystals was varied from those correspond to tetragonal, MPB and rhombohedral regions^32^. Three typical compositions from the corresponding regions with dimensions of 4 × 3 × 3 mm^3^ were selected for investigations. The compositions were determined to be 31PIN-37.2PMN-31.8PT, 25.4PIN-41.2PMN-33.4PT and 28.8PIN-33.3PMN-37.9PT by Energy Dispersive X-ray Spectroscopy. These crystals were designated as *A*, *B* and *C*, respectively. To compare the properties and structures of this new generation of crystals with previsouly reported binary system, a binary PMN-30PT crystal was also prepared into dimensions of 3 × 3 × 0.5 mm^3^, which was oriented along the [001] direction in its thickness. It is well-known to exhibit the highest piezoelectric and electromechanical properties for the binary systems^15^. All the ternary crystals were oriented along pseudocubic (100)/(010)/(001) planes, diced into rectangular parallelepipeds with dimensions of 4 × 3 × 3 mm^3^, and all the faces were polished to 0.25 *μ*m. In case of dielectric breakdown under application of high *E*, the corresponding rectangular parallelepipeds were then cut into several pieces along the longitudinal direction with thicknesses ranging from 0.5 to 1.5 mm. Note that these pieces had the same composition as the initial rectangular parallelepipeds due to a negligible composition gradient within such small crystals. The cut faces were successively re-polished perpendicular to the [001] direction to a roughness of 0.25 *μ*m using a polishing grinder. Prior to the property and structure measurements, all crystals were annealed at 800 K in air for 1 hour to release any pre-stress induced during the crystal growth and polishing.

### Dielectric and piezoelectric property measurements

Gold electrodes were deposited on the top and bottom surfaces by sputtering, then the macro-properties were measured along the thickness direction of the specimens. The temperature-dependent dielectric constant of crystals *A*, *B* and *C*, together with PMN-30PT, were characterized using a LCR meter (HP 4284A) in the zero-field-cooling condition. Unipolar strain vs *E* curves were measured in the temperature range of 300 to 420 K at 5 K temperature steps at a frequency of 1 Hz using a modified Sawyer-Tower circuit and linear variable differential transducer (LVDT) driven by a lock-in amplifier (Stanford Research SR850). The data was recorded under *E* = 50 kV cm^−1^ after at least two runs, thus the unipolar strain curves in Fig. 1(c–f) were recoverable (i.e., not a one-time effect from poling), and consistent with the way actuators are used. The specimens were poled under *E* = 10 kV/cm at 400 K for 15 mins, then cooled to 300 K. The piezoelectric coefficient *d*_33_ for various crystals were measured using a quasi-static Berlincourt *d*_33_ meter.

### X-ray diffraction

X-ray diffraction studies were performed using a Philips MPD high-resolution system. Facility details can be found in a previous publication^31^. Diffraction measurements for the ternary crystals were performed along pseudocubic (002) and (220) zones in the zero-field-heating condition from the annealed states. Line scans were performed over the corresponding angular regions with a 2*θ* step interval of 0.002°, whereas reciprocal-space mesh scans were taken with a 2*θ* interval of 0.005°. Studies were performed under electric field by coating gold electrodes on the appropriate faces, and attaching thin wires on the electroded sides using a small drop of conducting epoxy. Silicon grease with a high dielectric breakdown strength was used to prevent arcing. We designated the electroded faces as (001). First, to determine the phase transition under *E*, comprehensive line scans along the (00L) direction (i.e., electrodes was on top and bottom faces) were performed on the *A*-, *B*- and *C*-crystals at various temperatures by first increasing and subsquently decreasing *E*. The *E* was increased until arching or even dielectric breakdown occurred, or until a sharp single peak was observed that was insensitive to further increase in *E* (i.e., saturation state). At each temperature, measurements were began from the annealed condition. Upon finishing the measurements along (001), the electrodes were removed using a polishing blanket with 0.25 *μ*m aluminum powder. Then, the specimen was re-electroded on one pair of side faces perpendicular to the original (001) by sputtering. Thereafter, diffraction measurements were carried out along the (H00) and (HH0) zones. Next, to determine the domain configuration and stable phases, reciprocal-space mesh scans were obtained around the (200) Bragg peaks in the (H, 0, L) zone; and around (220) in the (H, H, L) zone. The mesh scans were recorded by a sequence of 2*θ*-*ω* scans at different *ω* offsets, and were shown in plots of intensity as a function of reciprocal lattice units (rlu). In this study, we fixed the value of rlu at a* = 2*π*/a = 1.550 Å^−1^, and all reciprocal-space mesh scans of PIN-PMN-PT were plotted in reference to this reciprocal unit.

## Additional Information

**How to cite this article**: Wang, Y. *et al*. Large field-induced-strain at high temperature in ternary ferroelectric crystals. *Sci. Rep*. **6**, 35120; doi: 10.1038/srep35120 (2016).

## Supplementary Material

Supplementary Information
