# Supplementary material for: Large field-induced-strain at high temperature in ternary ferroelectric crystals
Source: Sci Rep. 2016 Oct 13;6:35120. doi: 10.1038/srep35120 (PMC5062069; doi:10.1038/srep35120)
Supplement: Supplementary Information [file srep35120-s1.doc]

Supporting Information

Large Field-Induced-Strain at High Temperature in Ternary Ferroelectric Crystals

Yaojin Wang, Lijun Chen, Guoliang Yuan, Haosu Luo, Jiefang Li and D.Viehland


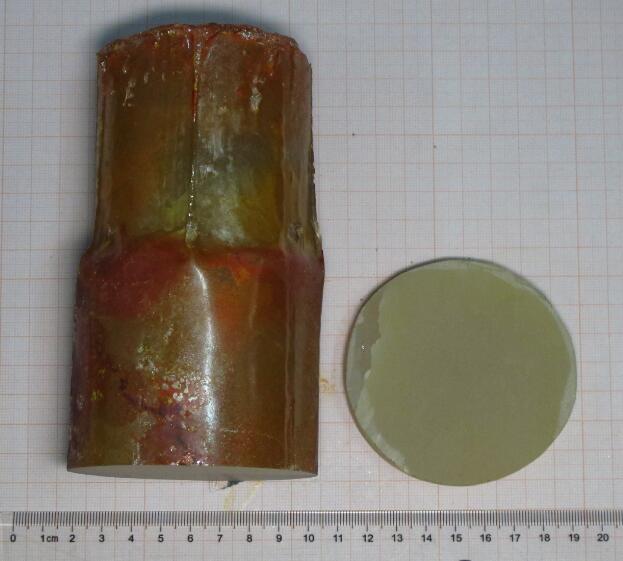


**Figure S1** Photographs for an as-grown large-size PIN-PMN-PT ternary crystal and [001]-oriented crystal wafer.


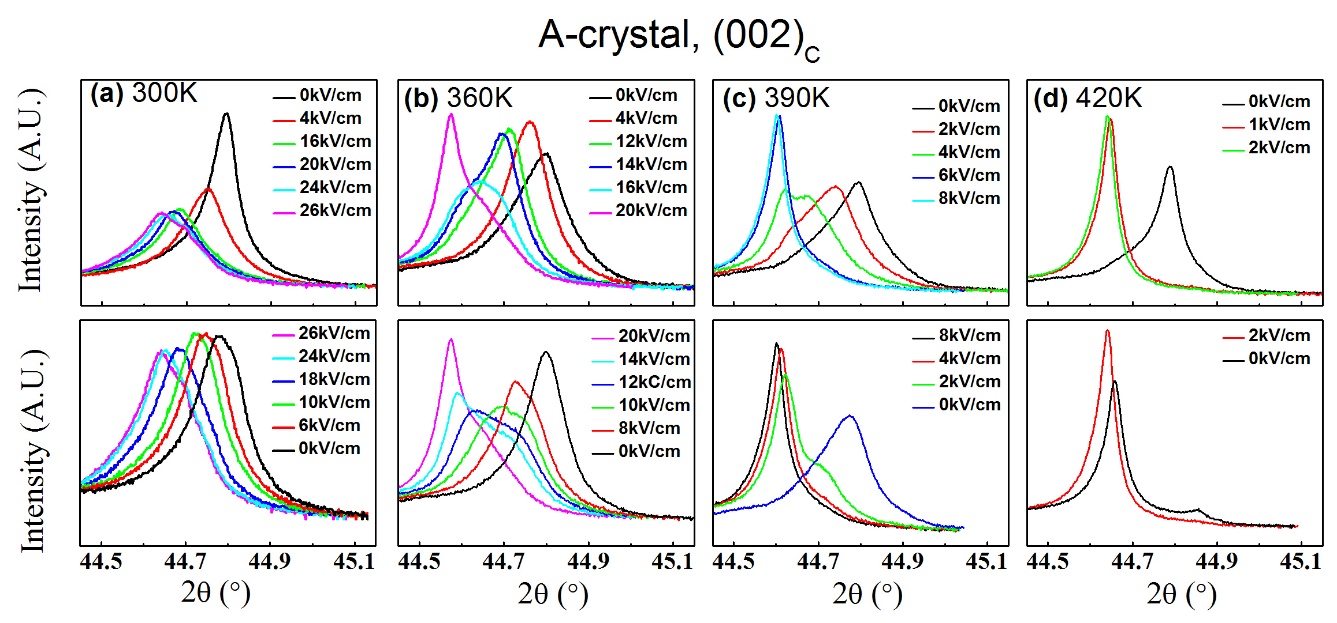


**Figure S2** Line scans along the pseudocubic (002) for *A*-crystals at various temperatures. The top and bottom panel present the evolution of diffraction peaks with *in situ* increasing and decreasing *E*//[001], respectively


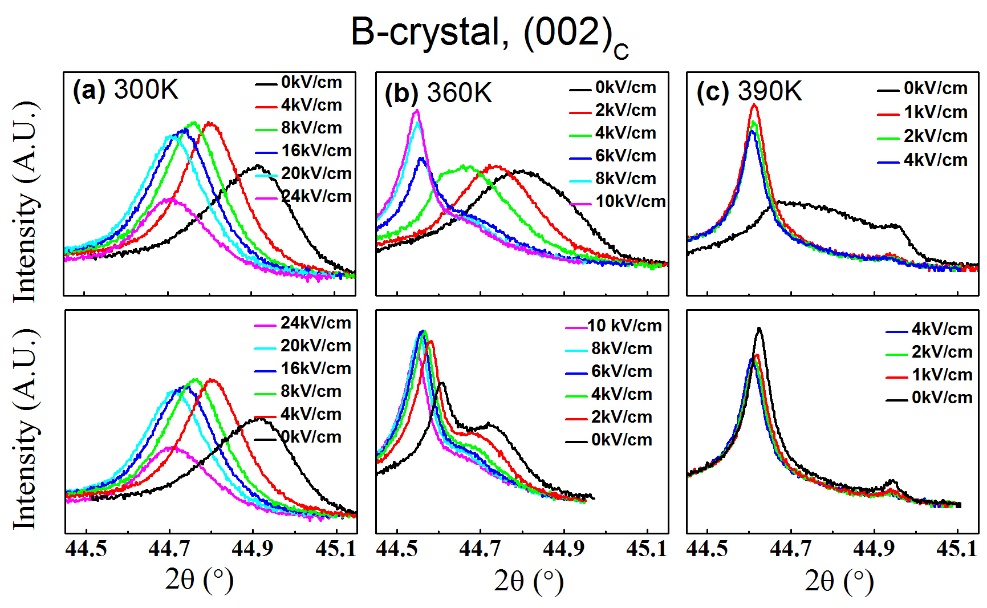


**Figure S3** Line scans along the pseudocubic (002) for B-crystals at various temperatures. The top and bottom panel present the evolution of diffraction peaks with *in situ* increasing and decreasing *E*//[001], respectively


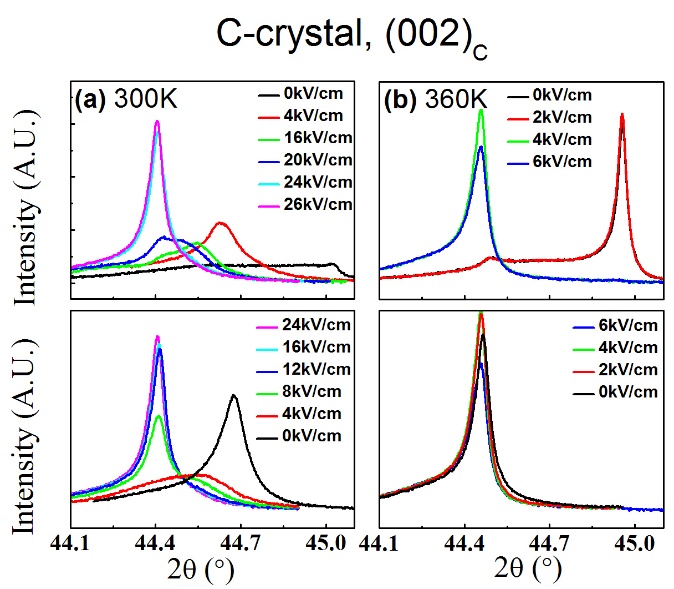


**Figure S4** Line scans along the pseudocubic (002) for C-crystals at various temperatures. The top and bottom panel present the evolution of diffraction peaks with *in situ* increasing and decreasing *E*//[001], respectively


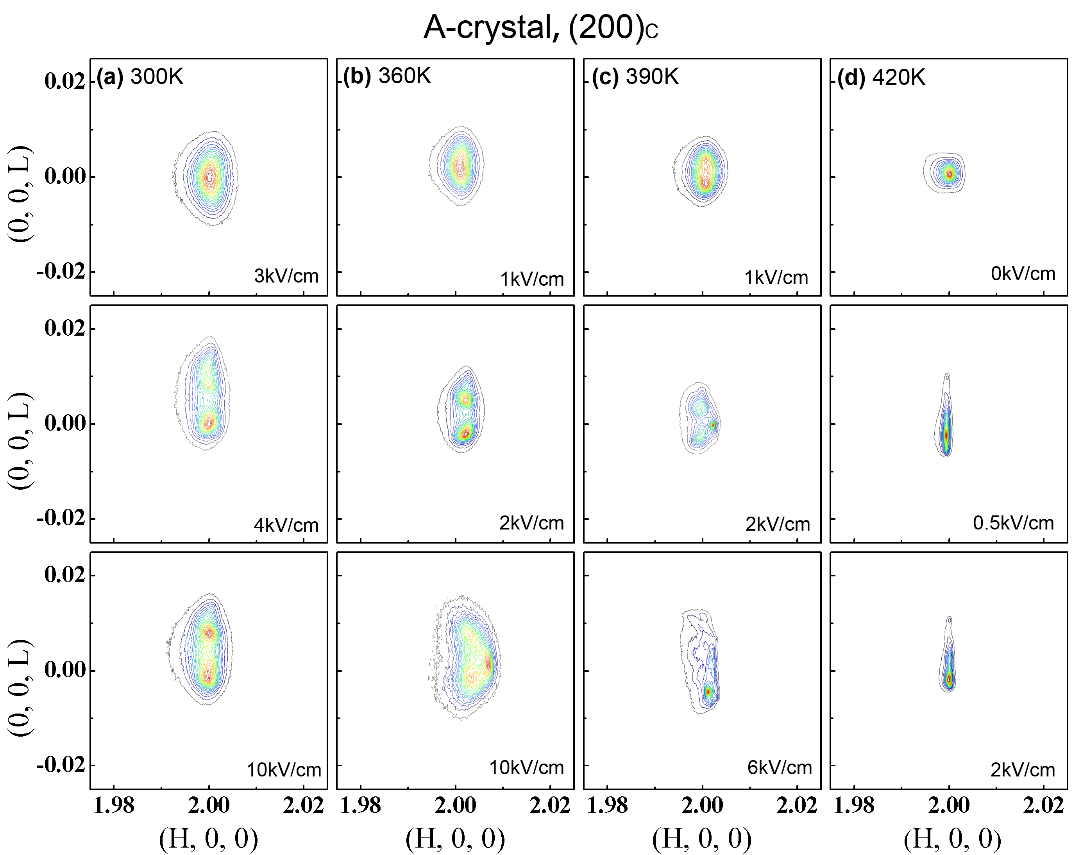


**Figure S5** Evolution of reciprocal-space mesh scans along the pseudocubic (200) zone for crystal A-crystal at various temperatures of (a) 300K, (b) 360K, (c) 390K and (d) 420K with *in situ* increasing *E*.


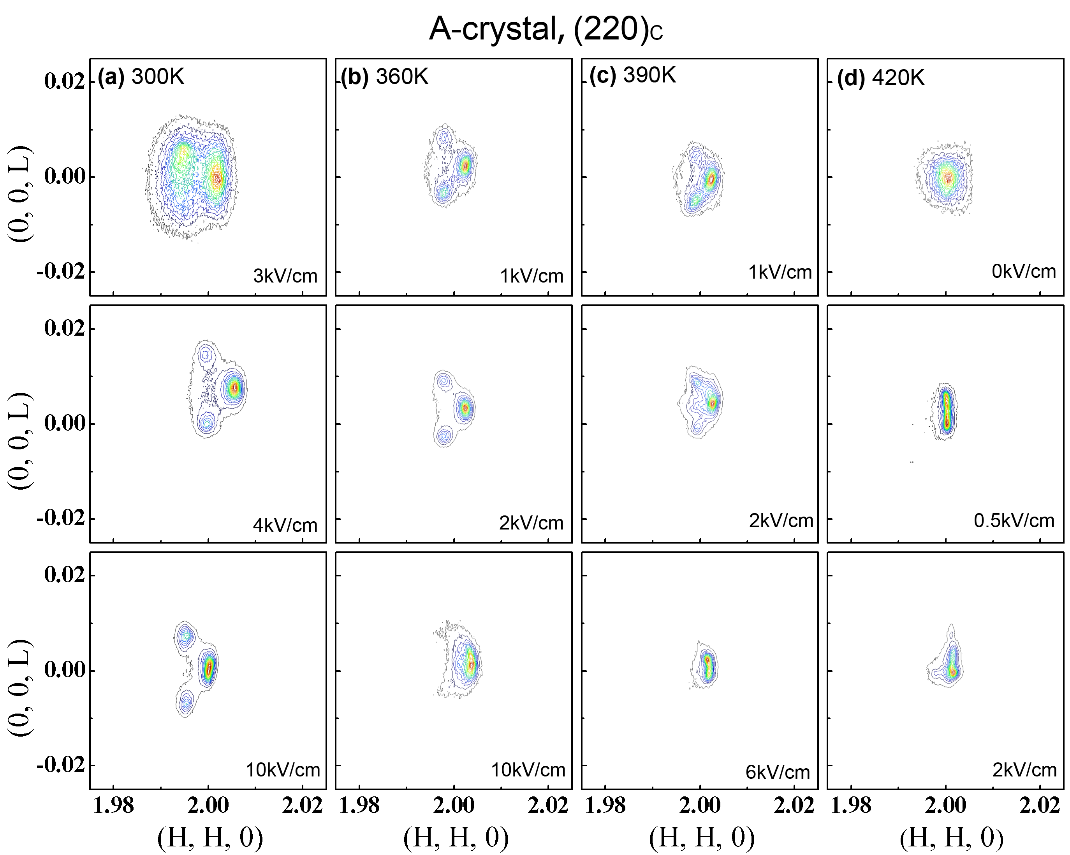


**Figure S6** Evolution of reciprocal-space mesh scans along the pseudocubic (220) zone for crystal A-crystal at various temperatures of (a) 300K, (b) 360K, (c) 390K and (d) 420K with *in situ* increasing *E*.


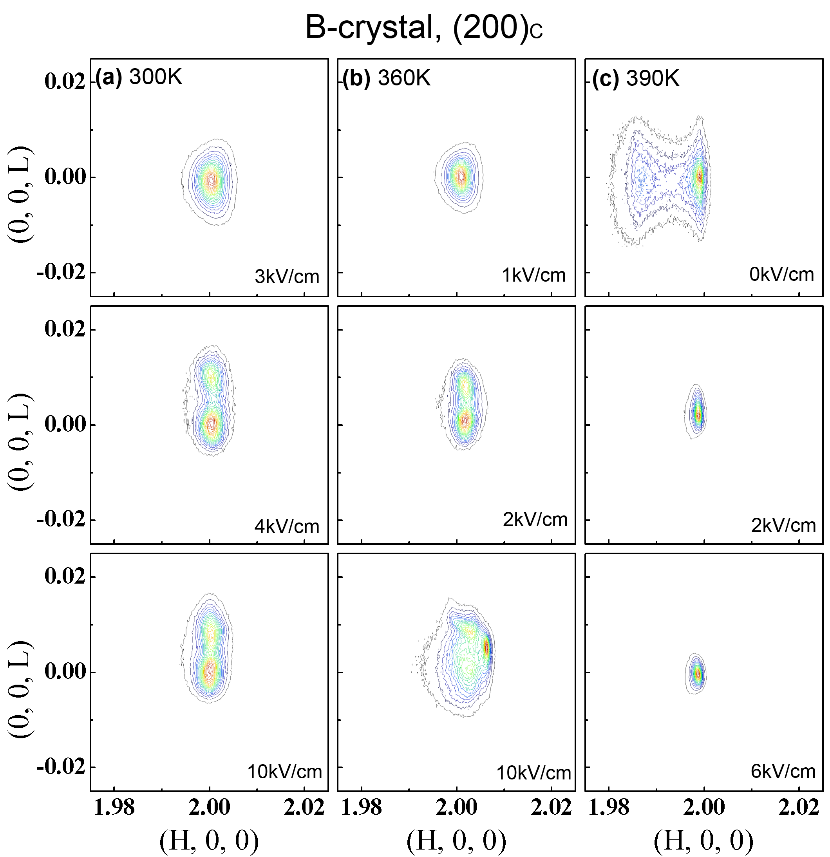


**Figure S7** Evolution of reciprocal-space mesh scans along the pseudocubic (200) zone for crystal B-crystal at various temperatures of (a) 300K, (b) 360K and(c) 390K with *in situ* increasing *E*.


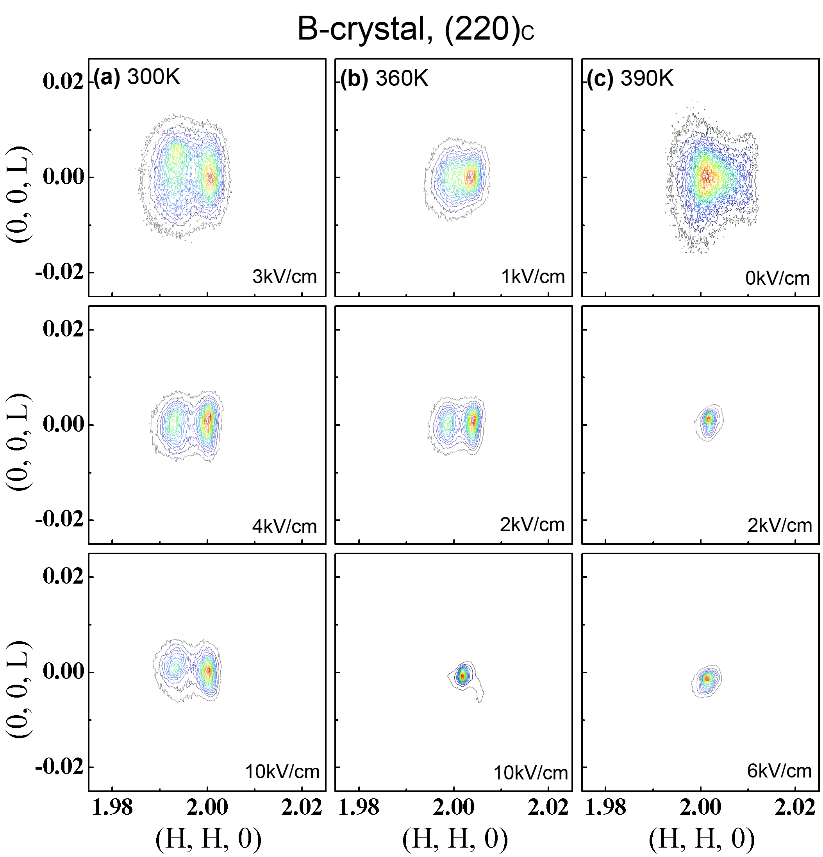


**Figure S8** Evolution of reciprocal-space mesh scans along the pseudocubic (220) zone for crystal B-crystal at various temperatures of (a) 300K, (b) 360K and(c) 390K with *in situ* increasing *E*.


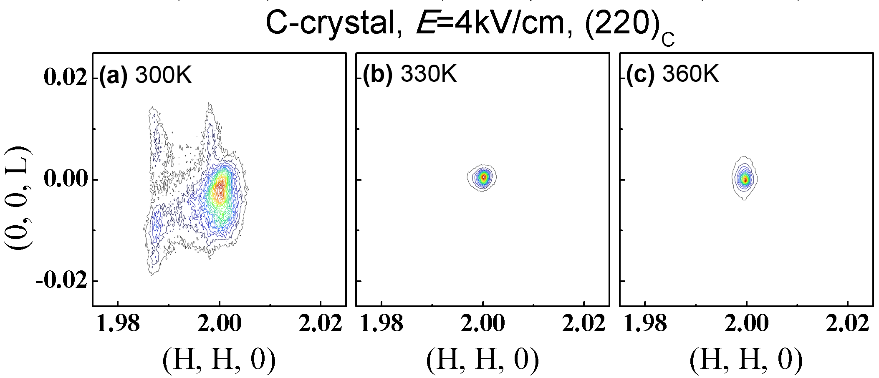


**Figure S9** Evolution of reciprocal-space mesh scans along the pseudocubic (220) zone for C-crystal at various temperatures of (a) 300K, (b) 330K and (c) 360K under *E*=4kV/cm.
